# Supplementary material for: Nonlinear optical encoding enabled by recurrent linear scattering
Source: Nat Photonics. 2024 Jul 31;18(10):1067–75. doi: 10.1038/s41566-024-01493-0 (PMC11449782; doi:10.1038/s41566-024-01493-0)
Supplement: Supplementary file 1 — Supplementary Sections 1–5 and Figs. 1–6. [file 41566_2024_1493_MOESM1_ESM.pdf]

nature photonics

Supplementary information https://doi.org/10.1038/s41566-024-01493-0

Nonlinear optical encoding enabled by recurrent linear scattering

In the format provided by the authors and unedited

Contents

1 Experimental setup 2

2 System stability 4

3 Nonlinear mapping approximated digitally by a multi-layer neural network 5

4 Mutual information calculation 7

5 More results from FashionMNIST reconstruction 8

References 9

1. Experimental setup

The experimental setup (Fig. S1) involves an off-the-shelf integrating sphere with a diameter of 3.75 cm. The sphere's interior is covered with a diffuse white reflective coating, creating a static scattering environment. There are three ports on the sphere. The first port, 8 mm in diameter, is attached a reconfigurable DMD that provides a dynamic scattering potential. This DMD, a Texas Instruments DLP9000X, is comprised of a grid of 2560 $\times$ 1600 micromirrors, each with a lateral dimension of 7.6 μ m and flipping between $+15°$ and -15°. The second port is for injecting light into the sphere. A continuous-wave, linearly polarized laser (Agilent 81940A) at a wavelength of 1550 nm is coupled to a single-mode fiber that feeds into this port. As the light enters the sphere, it undergoes multiple scattering events against the rough walls and the reconfigurable micromirrors. Light exits the sphere through the third port, which is 3 mm in diameter. The outgoing light is directed via a mirror to an InGaAs camera (Xenics Xeva FPA-640). Before reaching the camera, the light passes through a linear polarizer. The speckle intensity pattern associated with a specific configuration of the DMD is recored by the camera.

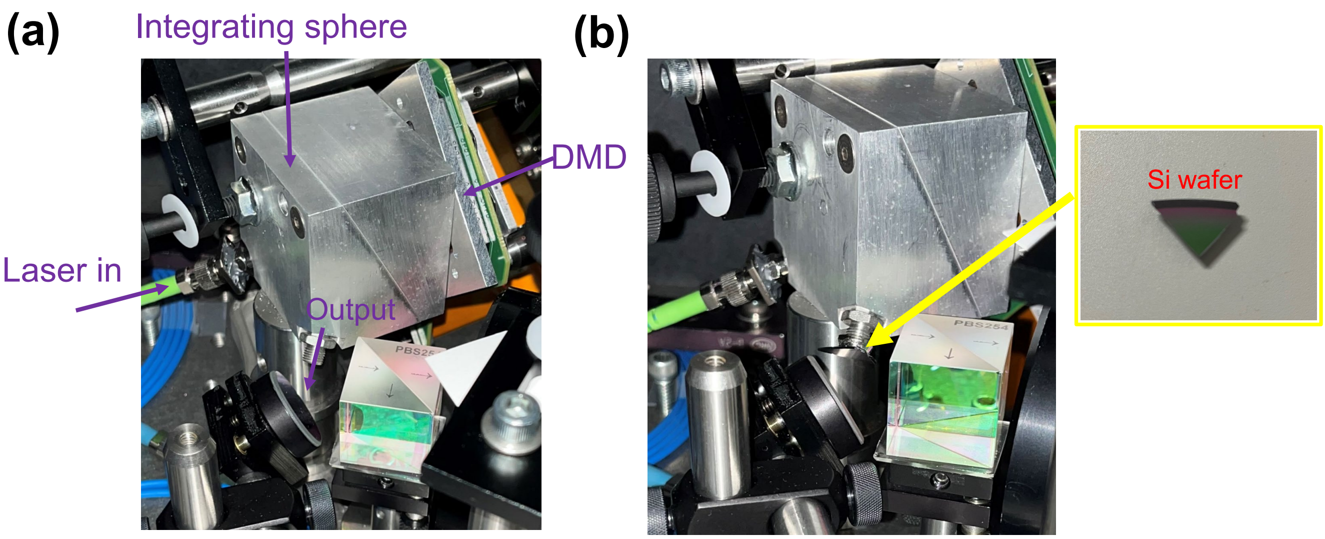

Figure S1. Photographs of our experimental setup showing key elements. (a) Our experimental setup includes a frequency-tunable continuous-wave fiber laser coupled to an integrating sphere via a single-mode fiber. The interior of this sphere has a rough reflectiving surface, providing a static scattering environment. A Digital Micromirror Device (DMD) is attached to one port of the sphere. The light from the laser, upon entering the sphere, undergoes multiple scattering by both the sphere's rough interior and the reconfigurable micromirrors of the DMD. Light escapes the sphere through a small aperture, and its intensity pattern is captured by a camera. (b) When the output port of the sphere is covered with a partial reflector – a silicon (Si) wafer, the dwell time of light inside the cavity increases, enhancing the chance of light reflecting off the DMD increases, and correspondingly the order of nonlinear mapping.

The micromirror array of the DMD is divided into macropixels of various sizes. Each macropixel has two states,

+1 and -1, corresponding to all its micromirrors being tilted by +15° and -15°, respectively.

At low input power (21 mW), despite multiple scattering events within the sphere, the output field is linearly related to the input field. However, the correlation between the DMD configuration and the output speckle pattern is nonlinear due to the multiple scatterings induced by the DMD. The experimental setup provides a nonlinear mapping between the output speckle pattern on the camera and the input pattern on the DMD. This mapping arises due to the multiple scattering of light by the DMD. It is this nonlinear relationship that forms the foundation for the passive nonlinear encoding technique we explore in this work. The DMD's capability to toggle the micromirrors between two positions, combined with the intricate scattering environment inside the integrating sphere, establishes a complex input-output relation. This complex relation can be intuitively understood as an innate deep nonlinear encoding process (see Fig. S3), which is the key element we investigate and exploit here.

In our study, the datasets utilized were predominantly 8-bit grayscale images, while the DMD operates in a binary state. To facilitate the loading of the input images into the DMD, we use a binary thresholding technique and utilized the Floyd-Steinberg dithering algorithm [1, 2] to spread the discretization error to neighboring pixels. This approach allow us to load images compatible with the DMD.

To control the scattering potential within our cavity, we employ two approaches aimed at modifying the number of scattering events occurring on the modulated area of the DMD. First, we reduce the number of scattering events by decreasing the modulated area of the DMD. This is achieved by shrinking the size of macropixels, resulting in a proportional decrease in the scattering potential originating from the input pattern on the DMD. Alternatively, we enhance the scattering events by increasing the dwell time of light inside the cavity. This can be done by covering the output port of the cavity with a partial reflector - a silicon wafer (0.63 mm thickness).

2. System stability

To characterize the stability of our experimental setup, we repeatedly measure the output speckle pattern (an example is shown in Fig. S2a) generated by the same pattern on the DMD and calculate its correlation with the speckle pattern taken at earlier time. More specifically, during data acquisition, we display the same DMD pattern (all micromirrors at +1 states) after taking several hundred input patterns and assess the speckle correlation with the initial speckle pattern, as depicted in Fig. S2b.

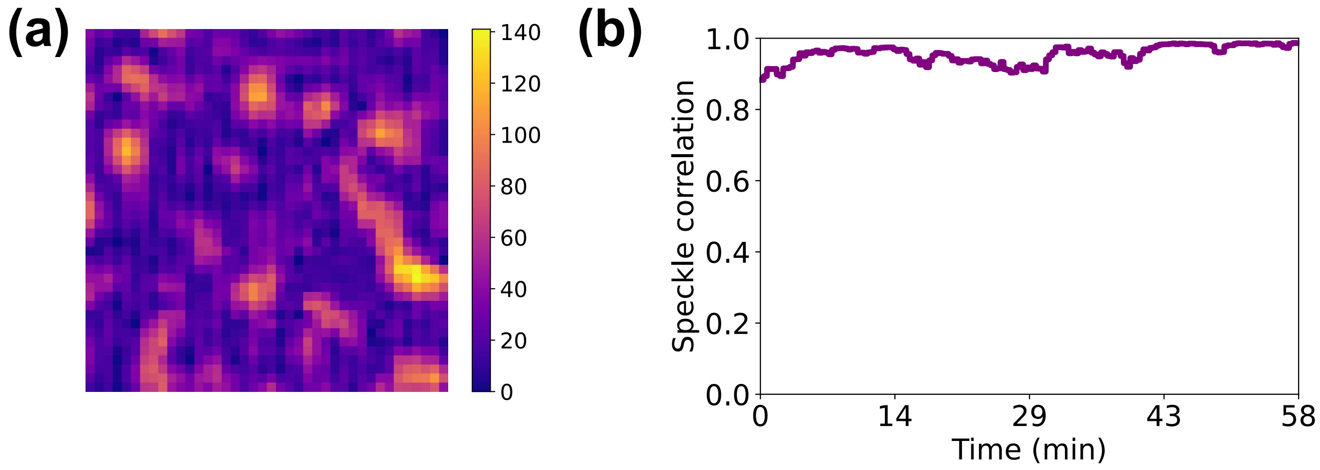

Figure S2. Characterization of system stability: (a) An example of a speckle pattern captured by the camera showing 64 speckle grains, which is captured when the output port of the cavity is covered by the Si wafer. (b) Correlation of output speckle patterns from the same DMD pattern, recorded over a duration of 58 minutes, within which all FashionMNIST data (in total 70,000 frames) were collected.

A high correlation value indicates that the scattering environment within the system remains consistent, providing evidence of system stability. Since we periodically measure the system's stability, we can set a threshold for the correlation value (e.g., 0.96) to filter highly correlated speckle patterns for further computations.

3. Nonlinear mapping approximated digitally by a multi-layer neural network

In order to examine the role of nonlinear random mapping in computing performance, we utilize a multilayer perceptron (MLP) neural network model to approximate these nonlinear mappings. The higher-order nonlinear random mapping instigated by multiple scattering in the cavity can be intuitively understood through a proxy - a deep neural network with weakly nonlinear activation functions, such as the rectified linear unit (ReLU) [3]. In such a network, higher-order nonlinearities accumulate as the activation function is applied across multiple layers. To quantitatively examine the multiple higher-order nonlinearities present in the nonlinear mapping introduced by multiple scattering in the multiple-scattering cavity, we train the MLP with fixed dimensions for input, hidden layers, and output, varying only the number of hidden layers (the depth). By maintaining an architecture constant in width while altering the depth of the network, we can isolate the effects of higher-order nonlinearities. This allows us to understand how changes in nonlinear random mapping can be interpreted as alterations in the depth of an equivalent deep neural network, thereby influencing the expressivity of the neural network.

Experimentally, we vary the size of the modulated area on the DMD, while fixing the number of macropixels on the DMD and the number of output modes (speckle grains on the camera). To train the MLPs, we display random patterns on the DMD and measure the output speckle patterns. Using these pairs, we determine the optimal network depth that provided minimal test loss. To achieve this, we perform a neural architecture search (NAS), keeping the width of the MLPs constant and allowing the architecture search space to vary only in terms of depth values.

For the training process, we use 5,000 input-output data pairs and selected the ReLU as the activation function. The ReLU is chosen due to its relatively weak nonlinearity, making it suitable for observing significant changes in depth. This selection allow us to discern more easily whether variations in the nonlinear order when altering the modulated area on the DMD can effectively be computationally approximated by neural networks of different depths.

As illustrated in Fig. S3, we can see that the number of depths in the optimal approximated MLP model increased with the modulated area on the DMD. This implies that a larger modulated area on the DMD requires the accumulation of more ReLU activation layers to approximate the nonlinear mapping, which in turn contributes to higher-order nonlinear random mapping.

Our findings suggest a strong correlation between the modulated area on the DMD and the depth required for best MLP approximation. This increased depth, resulting from the accumulation of ReLU activation layers, plays a crucial role in capturing the higher-order nonlinear mapping. This insight is valuable for understanding the underlying mechanisms of nonlinear mappings and the factors that influence the performance of neural networks in approximating such mappings.

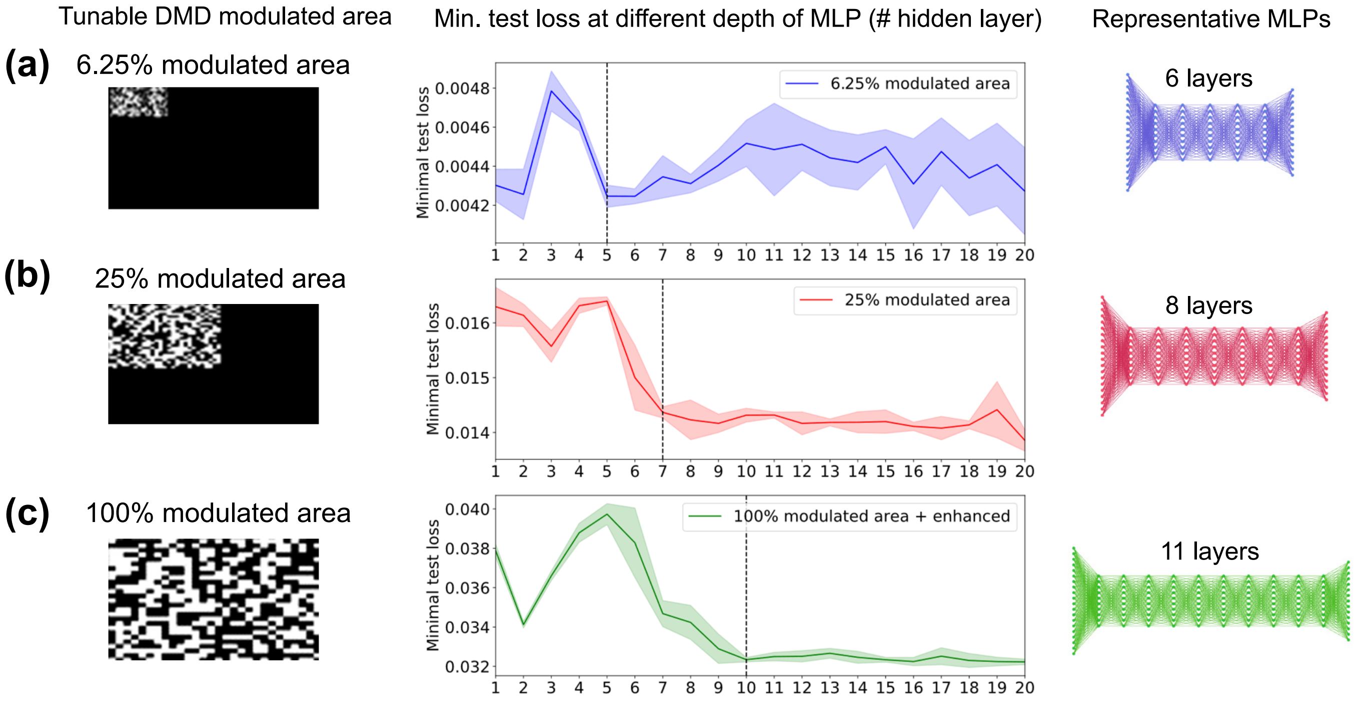

Figure S3. MLPs as proxies for nonlinear mapping from the DMD patterns to the output speckle patterns. Left column: modulated area on the DMD 6.25% (a), 25% (b), and 100% (c) of the entire DMD area covered by a partial reflector - a silicon wafer to enhance the scattering within the cavity. Middle column: The minimal test loss varies based on the number of hidden layers in the MLP approximation. Across 10 realizations, the minimal test losses are presented as the mean values (the solid lines) ± standard deviation (the shaded bands). Neural Architecture Search is employed to train and identify the most efficient neural network architecture for approximating the nonlinear mapping from the DMD pattern to the output speckle pattern. Right column: representations of equivalent MLPs with 6, 8, and 11 total layers for 6.25% modulated area, 25% modulated area, and 100% modulated area with a partial reflector - a silicon wafer added on the output port for enhanced scattering, respectively, demonstrating different depths of MLPs, with the number of hidden layers identified from the minimal optimal training loss.

4. *Mutual information calculation*

Mutual information serves as a key metric for gauging the common information shared between different random variables, finding broad utility in both the domains of physics and neural networks [4, 5].

In the realm of physics, mutual information facilitates the analysis of the interrelationships and interdependencies that exist among various physical systems or states. When applied to neural networks, mutual information offers insights to network functionality, the significance of various features, and the dynamics of the learning process, achieved through the quantification of shared information existing among inputs, outputs, or within different layers.

Here, mutual information is estimated using non-parametric methods based on entropy estimation from k-nearest neighbors distances. Given a dataset with continuous features X and a continuous target variable y, the mutual information between a single feature X_i and the target variable y_i can be estimated using dataset with continuous features X and target variable y. For each feature X_i in X: the entropy of feature X_i is $H(X_i) = -\sum_{x_i \in X_i} p(x_i) \log p(x_i)$, the entropy of target variable y is $H(y) = -\sum_{y_j \in y} p(y_j) \log p(y_j)$, and the joint entropy of feature X_i and target variable y is $H(X_i, y) = -\sum_{x_i \in X_i, y_j \in y} p(x_i, y_j) \log p(x_i, y_j)$. The mutual information between feature X_i and target variable y is $I(X_i; y) = H(X_i) + H(y) - H(X_i, y)$.

To estimate probability density functions, we use non-parametric methods like k-nearest neighbors. Using the dataset with continuous features X, target variable y, and parameter k, we compute the k-nearest neighbors distance for each point in X_i and y, find the k-nearest neighbors in X_i, and compute the distance $d_{X_i}(x_i)$ to the k-th nearest neighbor. For each point y_j in y, we find the k-nearest neighbors in y and compute the distance $d_y(y_j)$ to the k-th nearest neighbor. We estimate the entropies of X_i and y based on the distances $d_{X_i}(x_i)$ and $d_y(y_j)$, respectively:

$$H(X_i) \approx \psi(k) - \frac{1}{n}\sum_{i=1}^{n} \psi(n_x + 1) + \log \frac{\sum_{i=1}^{n} d_{X_i}(x_i)}{n} \tag{S1}$$

$$H(y) \approx \psi(k) - \frac{1}{n}\sum_{j=1}^{n} \psi(n_y + 1) + \log \frac{\sum_{j=1}^{n} d_y(y_j)}{n} \tag{S2}$$

where n_x and n_y are the number of neighbors within $d_{X_i}(x_i)$ and $d_y(y_j)$, respectively, and $\psi(\cdot)$ is the digamma function. The joint entropy $H(X_i, y)$ is estimated by computing the k-nearest neighbors distance in the joint space (X_i, y):

$$H(X_i, y) \approx \psi(k) - \frac{1}{n}\sum_{i=1}^{n} \psi(n_{xy} + 1) \tag{S3}$$

where n_{xy} is the number of neighbors within the k-th nearest neighbor distance in the joint space. We estimate the mutual information $I(X_i; y)$ using the entropy estimates:

$$I(X_i; y) = H(X_i) + H(y) - H(X_i, y) \tag{S4}$$

5. *More results from FashionMNIST reconstruction*

Here, we presented additional results from the FashionMNIST reconstruction task. Below are reconstructions from linear optical speckles with quadratic detection, utilizing an architecture-optimized 2-layer MLP as the decoder.

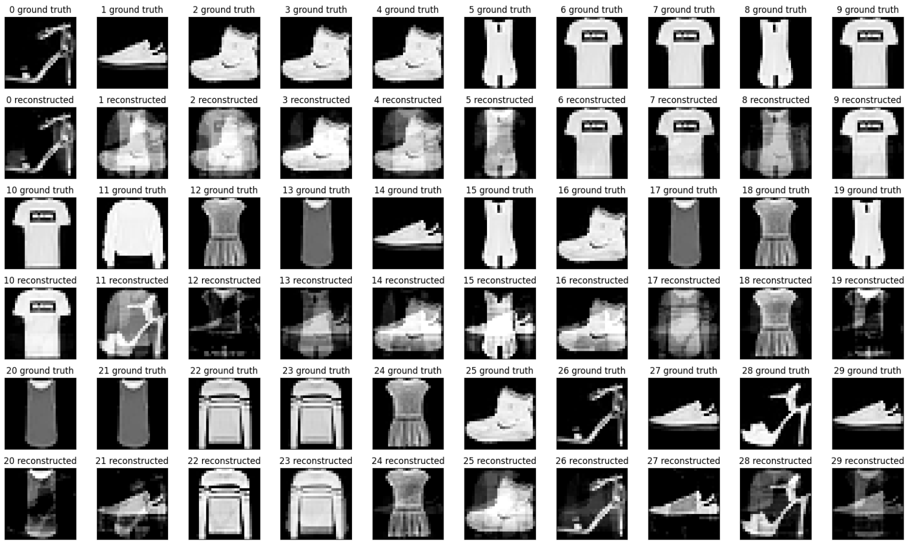

Figure S4. More reconstruction results are presented using linear optical speckle features (only 25 output modes) generated by the configuration shown in Fig. 3a. as optical encoder and with an architecture-optimized 2-layer MLP as a decoder.

We also presented additional results from the FashionMNIST reconstruction task. Below are reconstructions from nonlinear optical speckles with quadratic detection, utilizing an architecture-optimized 4-layer MLP as the decoder.

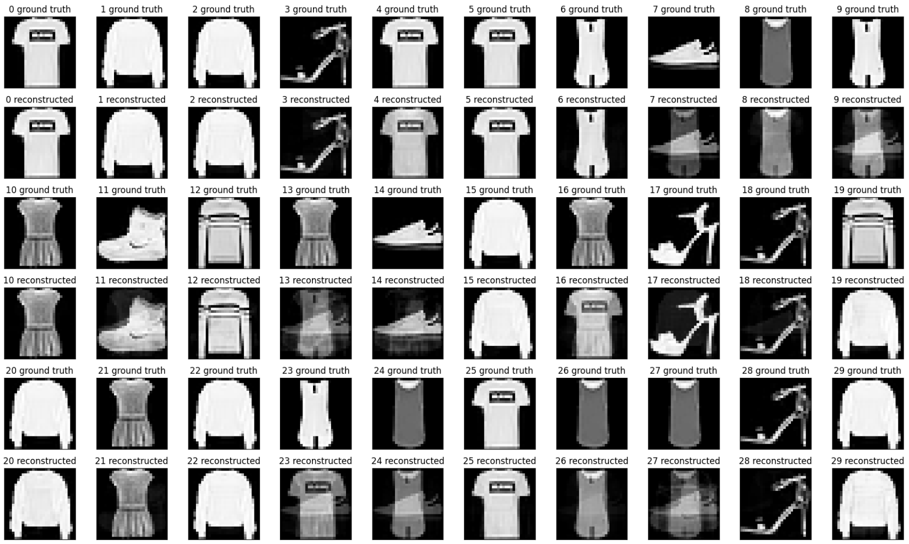

Figure S5. More improved reconstruction results from highly-nonlinear optical speckle features (only 25 output modes) generated by cavity configuration in Fig. 3c. as optical encoder and with an architecture-optimized 4-layer MLP as a decoder.

We further presented additional results from FashionMNIST reconstruction using nonlinear optical speckles with quadratic detection, employing the same decoder architecture (a 2-layer MLP) as with linear optical features with quadratic detection.

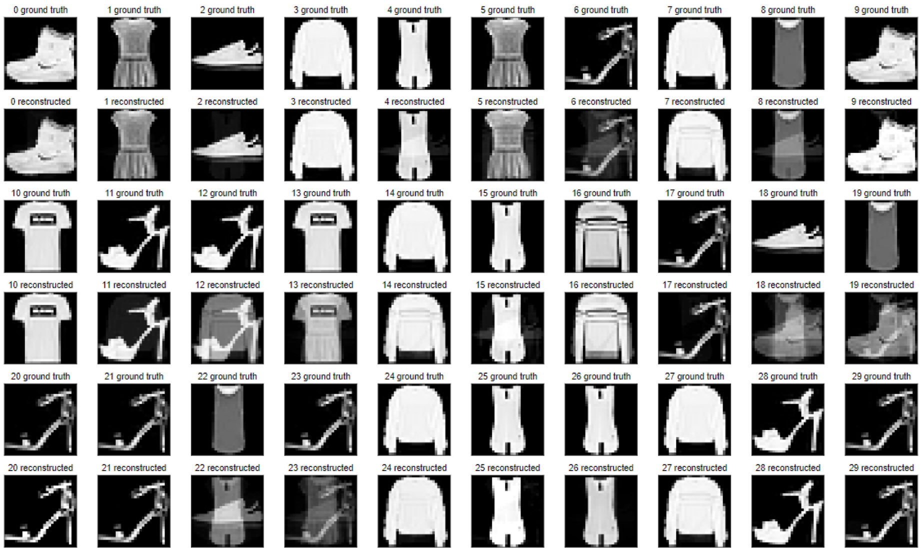

Figure S6. More improved reconstruction results from highly-nonlinear optical speckle features (only 25 output modes) generated by cavity configuration in Fig. 3c as optical encoder and with the same 2-layer MLP architecture used for Fig. S4 as a decoder.

[1] R. W. Floyd and L. Steinberg. *An adaptive algorithm for spatial greyscale.* In *Proceedings of the Society for Information Display*, pages 36–37, 1976.

[2] P. Heckbert. *Color image quantization for frame buffer display. ACM SIGGRAPH Computer Graphics*, 16(3):297–307, 1982.

[3] Vinod Nair and Geoffrey E. Hinton. *Rectified linear units improve restricted boltzmann machines.* In *Proceedings of the 27th International Conference on Machine Learning (ICML-10)*, pages 807–814, 2010.

[4] Thomas M. Cover and Joy A. Thomas. *Elements of information theory.* Wiley, 2012.

[5] Geoffrey Hinton, Li Deng, Dong Yu, George E. Dahl, Abdel-rahman Mohamed, Navdeep Jaitly, Andrew Senior, Vincent Vanhoucke, Patrick Nguyen, Tara N. Sainath, et al. *Deep neural networks for acoustic modeling in speech recognition: The shared views of four research groups. IEEE Signal processing magazine*, 29(6):82–97, 2012.
